# Re-designing nano-silver technology exploiting one-pot hydroxyethyl cellulose-driven green synthesis

**DOI:** 10.3389/fchem.2024.1432546

**Published:** 2024-08-14

**Authors:** M. Blosi, A. Brigliadori, S. Ortelli, I. Zanoni, D. Gardini, C. Vineis, A. Varesano, B. Ballarin, M. Perucca, A. L. Costa

**Affiliations:** ^1^ National Research Council of Italy, Institute of Science, Technology and Sustainability for Ceramics, (CNR-ISSMC), Faenza (RA), Italy; ^2^ National Research Council of Italy, Institute of Intelligent Industrial Technologies and Systems for Advanced Manufacturing (CNR-STIIMA), Biella, Italy; ^3^ Department of Industrial Chemistry “Toso Montanari”, Bologna, Italy; ^4^ Project HUB-360, Avigliana (TO), Italy

**Keywords:** green synthesis, silver nanoparticles, hydroxyethyl cellulose, clean technology, advanced antimicrobial nanocoatings

## Abstract

Re-designing existing nano-silver technologies to optimize efficacy and sustainability has a tangible impact on preventing infections and limiting the spread of pathogenic microorganisms. Advancements in manufacturing processes could lead to more cost-effective and scalable production methods, making nano-silver-based antimicrobial products more accessible in various applications, such as medical devices, textiles, and water purification systems. In this paper, we present a new, versatile, and eco-friendly one-pot process for preparing silver nanoparticles (AgNPs) at room temperature by using a quaternary ammonium salt of hydroxyethyl cellulose (HEC), a green ingredient, acting as a capping and reducing agent. The resulting nano-hybrid phase, AgHEC, consists of AgNPs embedded into a hydrogel matrix with a tunable viscosity depending on the conversion grade, from ions to nanoparticles, and on the pH. To investigate the synthesis kinetics, we monitored the reaction progress within the first 24 h by analyzing the obtained NPs in terms of particle size (dynamic light scattering (DLS), field emission scanning electron microscopy (FE-SEM), transmission electron microscopy (TEM)), Z-potential (ELS), surface plasmon resonance (UV-VIS), crystallographic phase (XRD), viscosity, and reaction yield (inductively coupled plasma-optical emission spectrometry (ICP-OES)). To explore the design space associated with AgHEC synthesis, we prepared a set of sample variants by changing two independent key parameters that affect nucleation and growth steps, thereby impacting the physicochemical properties and the investigated antimicrobial activity. One of the identified design alternatives pointed out an improved antimicrobial activity in the suspension, which was confirmed after application as a coating on nonwoven cellulose fabrics. This enhancement was attributed to a lower particle size distribution and a positive synergistic effect with the HEC matrix.

## 1 Introduction

Silver nanoparticles (AgNPs), known since ancient times as colloidal silver, find applications in different technologically relevant sectors, such as biomedical, optics, electronics, and catalysis (Shen et al., 2014; Dong et al., 2015; Haider and Kang, 2015; Khan et al., 2016; Gao et al., 2019; Ye et al., 2019; Rasmagin and Apresyan, 2020; Ardakani et al., 2021). They are extensively studied for their antibacterial, antiviral, and antifungal properties, making them optimal candidates for use as antimicrobial agents on different classes of materials, such as wound dressings, sanitary plastic materials, prostheses, materials for dental use, ceramics, textiles, and filters for water/air purifiers (Marini et al., 2007; Zhang et al., 2009; Chen et al., 2010; Mahltig et al., 2011; Quang et al., 2013; Brennan et al., 2015; Corrêa et al., 2015; Ibrahim, 2015; Atay, 2017; Moustafa, 2017; Ibrahim et al., 2018; Deshmukh et al., 2019; Ibrahim et al., 2019; Ibrahim et al., 2021; Gupta et al., 2022). The antimicrobial effect is exerted through multiple mechanisms, including disruption of bacterial cell membranes, interference with microbial enzyme systems, and generation of reactive oxygen species. This multifaceted mode of action reduces the likelihood of pathogens developing resistance compared to traditional antibiotics, which typically target specific cellular processes (Dakal et al., 2016). The numerous applications as coatings or additives in formulations have greatly increased the experimental (Gardini et al., 2018) and computational efforts (Furxhi et al., 2024) to identify design factors that can control their intended functionalities and safety profiles. Furthermore, in alignment with the European Green Deal program, the need for benign and biocompatible reagents forming stable suspensions over time represents a challenge to those concerned with the synthesis of colloids, offering a new approach for the one-pot synthesis of nanocomposites based on natural polymers as reducing and stabilizing agents. Replacing hazardous reagents with lower-impact ones is a new promising strategy that requires the chemical production sector to shift toward processes and technologies that need less energy, limit emissions, and minimize use of toxic reagents (Bhardwaj et al., 2020). In this context, the main requirements for successful industrial exploitation of the processes are based on scalability, which must balance the environmental impact with functionality. For colloidal nanosuspensions, combining high solid concentrations with long stability over time represents an added value toward sustainable upscaling; however, in literature, few methods ensure these requirements simultaneously (Tai et al., 2008; Srivastava et al., 2010; Hou et al., 2011; Tarannum et al., 2019; Yaqoob et al., 2020). In response to these needs, we present a patented green synthesis method for preparing AgNPs driven by a cellulose-derived compound acting on silver species both as a reducing and capping agent (Costa and Blosi, 2016). Since AgNPs enhance the antimicrobial functionality in synergy with their stabilizing and dispersing matrix (Morones et al., 2005; Cazzagon et al., 2022; Ameh et al., 2023), we have identified a quaternary ammonium salt of hydroxyethyl cellulose (HEC) as a key benign regent. HEC exerts intrinsic antimicrobial activity against a wide range of pathogens and is highly active in preventing bacterial resistance (Zubris et al., 2017; Kukushkina et al., 2021; Haktaniyan and Bradley, 2022).

HEC promotes the formation of AgNPs at room temperature without heating treatment. NPs are nucleated within the positively charged polymer. The method allows the one-shot preparation of spherical NPs of approximately 15–20 nm diameter in the form of a stable concentered hydrogel (0.5% wt) with tunable viscosity, overcoming the limitations of known methods, while satisfying the requirements for a future industrial scale-up. We also present a deep investigation of the synthesis process by monitoring the reaction progress within the first 24 h and evaluating how the main synthesis parameters affect both physicochemical and antibacterial properties. The potentialities of this method stem from its combined characteristics of simplicity, low environmental impact, reproducibility, and versatility, making the prepared material of great industrial interest as an antimicrobial coating that is easily applicable to numerous strategic sectors (Marassi et al., 2018; Cazzagon et al., 2022).

Further quantitative investigations in the proposed nano-silver technology were aimed at assessing the effect of different synthesis options on the physicochemical features of obtained nano-silver products and identifying the combination of the synthesis variables that allow simultaneously maximizing the nano-silver product antibacterial performance level, while assuring a safer and more sustainable synthesis route. The identified solution, consistent with the safe and sustainable by design approach, was obtained by implementing the multi-criteria decision analysis algorithm by using the MultiOptimal™ IT platform (https://www.projecthub360.com/multioptimal360/).

## 2 Experimental procedure

### 2.1 Chemicals

For the preparation of AgNPs, the following analytical-grade reagents were used: AgNO_3_, NaOH (Sigma-Aldrich, United States), and a quaternary ammonium salt of hydroxyethyl cellulose, SoftCAT SL-30 Polymer (INCI Name: Polyquaternium-67), purchased from Dow Chemical, United States, hereafter named as HEC. This polymer, primarily used in hair and skin cleansing/conditioning products or as a moisturizer, is used here as both a capping and reducing agent for AgNPs. HEC is characterized by cationic molecules possessing a positive charge at the nitrogen atoms balanced by chloride counterions (Supplementary Figure S1) (Pfau et al., 1997; Ballarin et al., 2011).

### 2.2 Synthesis procedure

HEC (1.47 g) was dissolved in 18.0 mL of water by mechanical stirring, and 8.0 mL of the Ag salt precursor solution (AgNO_3_, 0.2 M) was added. The presence of silver in the solution containing chloride ions coming from HEC produced a whitish color due to the formation of silver chloride. After 1 min, 4.0 mL of a NaOH solution (1.0 M) was poured and stirred for 5 min. The obtained suspension turned brown/yellow, suddenly increasing its viscosity in the form of the hydrogel. The product was kept in dark conditions at room temperature for 48 h, promoting the total reduction of Ag^+^→AgNPs and reorganization of the gel into a low-viscosity suspension with a high concentration of AgNPs (0.5% wt).

To explore the design space and identify the most effective AgHEC-based solutions, we modified the main stoichiometric ratios in terms of HEC/Ag and NaOH/Ag by preparing five variants at different molar ratios.

Along with the formation of AgHEC, cellulose (HEC) hydrolysis occurs, as indicated by the decrease in the viscosity of the gel during the first 12–48 h. Typically, the synthesis involves NaOH/Ag and HEC/Ag molar ratios of 2.8 and 5.5, respectively, and a silver nitrate concentration of 0.05 M (0.5% wt of metal). In exploring the design space, the HEC/Ag molar ratio varied from 5.5 to 1.4, and the NaOH/Ag molar ratio varied from 4.2 to 1.4.

### 2.3 Application of AgHEC on nonwoven cellulose fabrics

AgHEC compounds were applied to nonwoven cellulose samples (140 g/m^2^, dimensions of 100 mm × 50 mm) by using a dip-coating deposition process. We set up a dipping/withdrawing speed of 2 mm/s and a soaking time of 5 s into the prepared Ag-based suspensions, previously diluted at a metal concentration of 0.01% wt. After drying at room temperature for 1 h, the coated samples were treated in the oven for 10 min at 90°C.

### 2.4 Analytical characterization

Prepared nanosols were characterized by optical spectroscopy (UV–VIS), dynamic light scattering (DLS), X-ray diffraction (XRD), inductively coupled plasma-optical emission spectrometry (ICP-OES), viscosity, and field emission electron microscopy (FE-SEM).

UV–VIS extinction spectra were measured using a Lambda 35 Spectrophotometer (PerkinElmer, United Kingdom), by using a quartz cuvette as a sample holder. Samples were prepared by diluting the as-prepared colloidal suspension at the same metal concentration (6–12 mg/L).

DLS was used to monitor the hydrodynamic diameter and particle size distribution of the suspensions. Measurements were carried out using Nano S (Malvern, United Kingdom), working at a fixed angle of 173°. Samples were properly diluted with water and poured into a polystyrene cuvette before measurement. The hydrodynamic diameter includes the coordination sphere and the species adsorbed on the particle surface, such as stabilizers and surfactants. A polydispersity index (PDI) ranging from 0 to 1 and quantifying the colloidal dispersion degree was also provided; for a PDI below 0.2, a sol can be considered monodispersed.

Diffraction patterns were collected on the synthesized samples dripped on a glass slide and dried at 80°C for 15 min. Analyses were performed by using the Bruker D8 Advance Diffractometer (Germany) operating in the θ/2θ configuration with a LynxEye detector (acquisition condition 20°–80° 2θ range, 0.02 step size, and 16 s time per step equivalent). To derive the mean crystallite size, we implemented the Debye–Scherrer equation by using the following parameters: K = 0.9 and λ = 1.54056 Å.

Unreacted metal cations were detected by ICP-OES (5100 vertical dual-view apparatus, Agilent Technologies, United States) to infer the reaction yield. The percentage ratio (Ag^+^/Ag) was assessed after filtering 10.0 mL of the suspension using an ultra-centrifugal filter (UCF) unit (Amicon Ultra-15, 10 kDa, Millipore) at 5,000 rpm for 30 min by retaining AgNPs and removing Ag^+^ ions.

Coated fabrics were soaked for 5 min in 6.0 mL of a solution 50:50 v/v of HNO_3_ (65% v/v Sigma Aldrich) and H_2_O_2_ (30% v/v Sigma Aldrich), then rinsed with 6.0 mL of MilliQ water, and analyzed by ICP-OES to quantify the mass of applied Ag onto the fabric and expressed as micrograms of the metal on grams of the substrate.

The viscosity was evaluated to monitor the change in the polymerization degree of HEC, which occurs simultaneously with the formation of AgNPs embedded into the hydrogel. Viscosity tests were performed by rotational viscometry at 25°C by using a rheometer (Bohlin C-VOR 120, Bohlin Instruments, United Kingdom) equipped with a cone and plate geometry with a cone angle of 4° and a diameter of 40 mm (CP4/40) and controlling the temperature using an external water thermostat (Julabo KTB-30). The shear viscosity was measured by applying a constant shear rate of 0.1 s^−1^ on samples withdrawn from the reaction environment at different times (to a maximum of 8 h).

Ag nanoparticles were observed both using a field emission scanning electron microscope (Supra 40, Zeiss, Germany) and a transmission electron microscope (FEI Tecnai F20).

For FESEM analysis, specimens were prepared by dripping the colloidal suspensions on an aluminum stub. Specimens were prepared by dripping the colloidal suspensions, previously diluted in water (Ag concentration of 0.002% wt), onto a metal grid coated with a polymer film. Drops were evaporated at room temperature in the ambient atmosphere. Image analysis was performed on more than 150 particles for each sample to calculate particle size distribution and mean diameter. Scanning transmission electron microscopy (STEM) pictures were recorded by using a high-angle annular dark field (HAADF) detector. Image analysis was performed on more than 300 particles for each sample to calculate the particle size distribution and mean diameter.

For TEM analysis, Ag suspensions diluted at 0.005% wt were drop-cast on a holey carbon film supported by a gold grid. The specimens were then dried at 50°C to remove the solvent. TEM images were collected in the phase contrast mode using a selected area electron diffraction (SAED) device operating at 200 keV. Image analysis was performed on more than 400 particles.

### 2.5 Antibacterial characterization of AgHEC-based suspensions

Tests were carried out in compliance with the EN 1040:2005 standardized method against *Escherichia coli* (ATCC 10536) and *Staphylococcus aureus* (ATCC 6538). Suspensions at concentrations ranging from 50.0 to 0.05 mg/L were tested at four dilutions for a contact time of 24 h. Each result is the average of three independent measurements. Blank solutions (identified as HEC-01, HEC-02, HEC-04, HEC-05, and HEC-06) were considered a comparison and were prepared by using the procedure of the corresponding Ag suspension without adding the metal. Antibacterial results are reported on a logarithmic scale.

### 2.6 Synthesis process safety and sustainability assessment

In the framework of safe and sustainable by design processing to obtain chemicals and materials with addressed functionality, multiple performance indicators have been addressed in association with the synthesis of AgHEC nano-silver products.

The sustainability of the synthesis route was assessed by applying the life cycle assessment (LCA) methodology according to the ISO 14040-44 standard by applying a “cradle to gate approach.” The functional system has been identified as the AgHEC synthesis process. The defined functional unit (FU) is the amount of 30 g of AgHEC hydrogel product obtained via the synthesis process. Energy–mass balance analysis has been performed by considering raw materials and energy inputs to the system and inventorying associated system emissions to the environment. The impact assessment was conducted by applying the CML 2001 impact assessment method. The source of secondary data employed is the ecoinvent 3.7 database.

Parallel to the LCA, the life cycle costing (LCC) analysis has been done to assess the economic implications of the addressed synthesis options investigated. Reagents, energy, operators, equipment, and infrastructure use represent the main cost drivers implied in the synthesis process and are considered in the analysis.

### 2.7 Identification of the multioptimal options for safe and sustainable AgHEC synthesis

The goal of designing a safe and sustainable synthesis process to produce AgHEC was pursued by implementing a multioptimization algorithm based on multi-criteria decision analysis (MCDA), which allows for identifying the candidate design cases, which simultaneously maximizes multiple performance indicators. The requirement for running the multioptimization algorithm is the availability of a harmonized dataset derived by experimentation (physicochemical characterizations and antibacterial performance measurement) and modeling and computation (LCA and LCC data), which implies obtaining attribute performance and levels associated to the point of the DoE matrix. MultiOptimal360™ is the IT platform employed to run the multioptimization algorithm. The MultiOptimal360™ workflow starts from the input of the case study-specific data: a) the key decision factors (KDFs), set of data for the reference design cases in the DoE matrix; b) the associated key performance indicators (KPIs), data obtained by experimental assessment and modeling; and c) physicochemical characterization data related to the synthesized NPs, representing the reference synthesis design cases. The implementation of the MCDA algorithm allows the selection of the multioptimal design cases that simultaneously meet the design criteria. For the specific AgHEC safe and sustainable synthesis design case study, these criteria include minimizing (1) the climate change environmental impact parameter (GWP), (2) the human toxicity potential (HTTP), and maximizing (3) the compound functional cost performance indicator, aiming at achieving minimum cost and maximum antimicrobial functionality for the specific bacterial strains.

The multioptimization computation resolution was varied from n.20 points to n.50 points per unit KDF variability range in the design space. MultiOptimal360™ through the MCDA algorithm selected the points in the design space (representing specific combinations of KDF values), which are associated with the KPI values that comply with the set design criteria. This allowed restricting all possible design case options (all design space points) to a subset of candidate cases that are safe and sustainable by design cases (represented by the design space and points lying only on the multi-optimal curve). The final selection criteria were based on choosing the least environmental impact and safest (least toxic) synthesis process, compliant with an acceptable compound functionality performance level. Points in design space outside the multioptimal curve fail to simultaneously minimize environmental and cost impacts, human toxicity, and to maximize antimicrobial functionality.

### 2.8 Antibacterial characterization of nonwoven cellulose fabrics

The antimicrobial activity was evaluated on coated substrates, approximately 0.5 g, according to ASTM E 2149-01, “Standard test method for determining the antimicrobial activity of immobilized antimicrobial agents under dynamic contact conditions.” The method is designed to evaluate the resistance of non-leaching antimicrobial-treated specimens to the growth of microbes under dynamic contact conditions. The applied bacteria are *Escherichia coli* ATCC 11229 (Gram-negative) and *S. aureus* ATCC 6538 (Gram-positive). The incubated test culture was diluted to give a concentration of 1.5–3.0 × 10^5^ CFU/mL (working dilution). Each sample was transferred to a flask containing 25.0 mL of the working dilution. All flasks were shaken for 1 h at 190 rpm. After a series of dilutions, 1.0 mL of the solution was plated in nutrient agar. The inoculated plates were incubated at 37°C for 24 h, and surviving cells were counted. The antimicrobial activity was expressed in % reduction of the organisms after contact with the test specimen compared to the number of bacterial cells surviving after contact with the control. Each result represents the average of three independent measurements.

## 3 Results and discussion

### 3.1 Hypothesis of reaction

**FIGURE 1:**
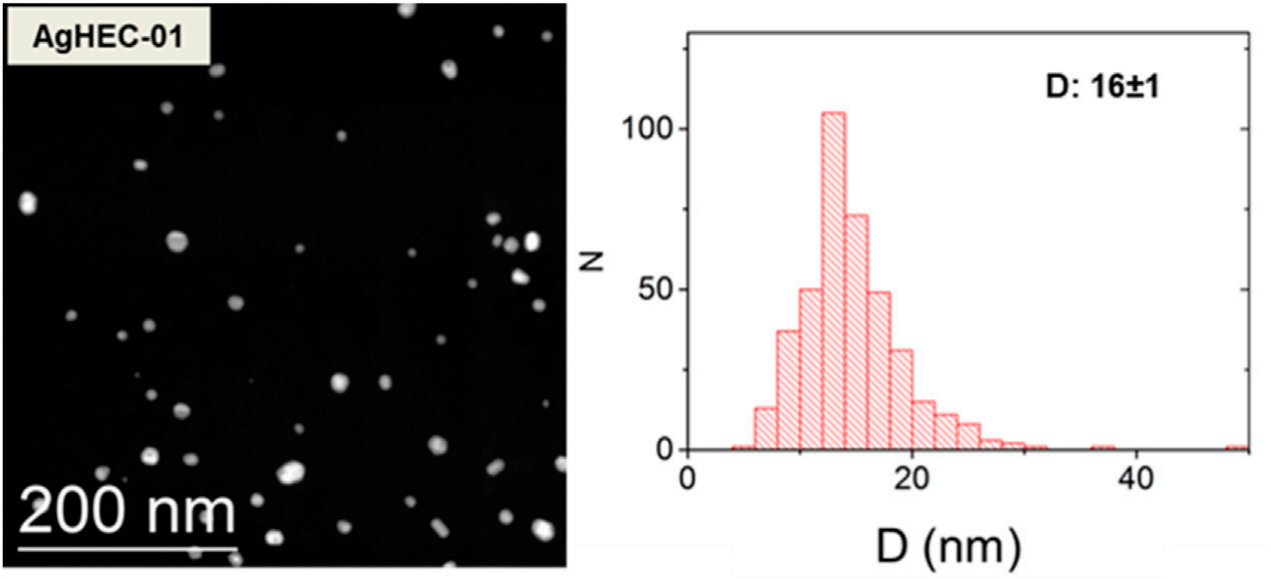
TEM image and corresponding particle size distribution measured on more than 400 NPs for AgHEC-01.

The hydroxyethyl cellulose-based compound acts both as a chelating and reducing agent toward the silver ions, forming a colorless, high-viscosity gel after the addition of NaOH into the reaction path. When the alkaline solution is added, the suspension immediately turns into a gel, initially assuming a dark brown color due to the formation of the first Ag nuclei, and after approximately 24 h, the color changes to brown-yellow, indicating the complete formation of Ag nanoparticles. Typically, wet synthesis of noble metals must ensure the optimal concentration of fundamental reactants: a reducing agent promoting the transformation of metal ions into their zero valent forms, a chelating agent preventing the excessive particle growth, and a catalyst triggering the reduction, particularly in the presence of weak reducing agents. As reported by our previous publications (Blosi et al., 2014; Blosi et al., 2016) dealing with the preparation of noble metal NPs through glucose, used as a weak reducing agent, the reduction power of glucose is drastically enhanced in alkaline conditions (Watanabe et al., 2005). The alkaline environment created by adding sodium hydroxide promotes α-proton dehydrogenation, causing the glucose ring to open and form an aldehyde group that can reduce the metal ions and finally oxidize to gluconic acid. Similarly, for AgHEC, we can hypothesize that sodium hydroxide promotes the generation of new reducing aldehydic end groups in HEC macromolecules (El-Sheikh et al., 2013), enabling the reduction of Ag ions that are nucleated and grown in the form of nanoparticles embedded in the polymer matrix, which also acts as a chelating agent. Therefore, an optimal balance among reduction, nucleation, and growth phenomena is fundamental to ensuring total Ag^+^ conversion, particle growth control and size homogeneity, and colloidal stability over time. AgHEC-01 corresponds to the standard procedure, optimized based on colloidal stability over time, and synthesized by applying the following molar ratios: HEC/Ag = 5.5; NaOH/Ag = 2.8 and successfully tested against SARS-CoV-2 (Costa et al., 2022). The so-prepared Ag nanoparticles embedded in the hydrogel, as observed by TEM analysis, appeared as spheroidal particles. With a confidence interval of 99%, an average dimension of 16 ± 1 nm was assumed (Figure 1). DLS measurements indicated a hydrodynamic diameter in the 250–260 nm range due to the steric hindrance induced by the cellulosic matrix chelating the nanophases. A summary of the collected physicochemical data is provided in Table 1.

**TABLE 1:** Effect of HEC/Ag and NaOH/Ag molar ratios on the main physicochemical properties of AgHEC samples at t = 24 h.

Sample	[HEC/Ag]	[NaOH/Ag]	TEM (nm)	ØDLS (nm)	PDI	Z-potential (mV)	pH	λ_max_ (nm)	Ag yield* (%wt)
AgHEC-01	5.5	2.8	16 ± 1	286 ± 4	0.3 ± 0.04	16.5 ± 0.3	10.3	415 ± 4	100
AgHEC-02	2.8	2.8	18 ± 1	174 ± 1	0.3 ± 0.01	11.5 ± 0.4	10.5	408 ± 4	100
AgHEC-03	1.4	2.8	n. a	3,280 ± 370	0.8 ± 0.14	3.9 ± 0.2	11.4	424 ± 4	62
AgHEC-04	2.8	1.4	17 ± 1	219 ± 1	0.3 ± 0.04	15.2 ± 0.1	9.3	409 ± 4	82
AgHEC-05	2.8	3.7	18 ± 1	204 ± 2	0.2 ± 0.01	11.3 ± 0.3	10.8	408 ± 4	100
AgHEC-06	6.4	1.4	13 ± 1	343 ± 9	0.3 ± 0.01	20.1 ± 0.4	9.6	406 ± 4	100

All samples have been synthesized with Ag conc = 0.05 M (0.5% wt). *, calculated as the Ag^+^/Ag ratio.

The positive value of Z-potential was consistent with the presence on the nanoparticles’ surface of the cationic hydroxyethyl cellulose. The surface plasmonic resonance (SPR) band evidenced an absorption wavelength of 415 nm, consistent with the typical wavelength range (380–460 nm) of spherical AgNPs (Kumbhar et al., 2005; Wilcoxon and Abrams, 2006; Alzoubi et al., 2023). To monitor the nanoparticles’ nucleation and growth evolution, we sampled the reaction environment at increasing reaction times and analyzed the suspension by FE-SEM, UV–VIS, and XRD (Supplementary Figures S2–S4). Because of the smaller resolution capacity of FE-SEM than TEM, we derived slightly larger mean diameters. Nonetheless, the data allowed us to capture the evolution of particle size over the course of the reaction. The study was carried out on AgHEC-01, prepared in agreement with the standard procedure. FE-SEM images reported in the Electronic Supplementary Material (Supplementary Figure S2) showed, at the beginning of the reaction, time 0 (t = 0), a low particle density, with the first nuclei identified as spheroidal particles surrounded by cellulose agglomerates (the biggest whitish domains) and a mean diameter of 12 ± 5 nm (Supplementary Figure S2A). For this sample, we detected an SPR band at 407 nm (Supplementary Figure S3), consistent with the formation of nanosized Ag particles. The weak band intensity was probably due to the low density of nanoparticles and the large number of newly formed nuclei with a diameter less than 5 nm, which were not detectable by FE-SEM. An almost unchanged situation was highlighted by the UV–VIS spectrum of sample AgHEC-T1 registered after 1 h (Supplementary Figure S2B), as confirmed by a mean diameter placed at 13 ± 5 nm and a weak SPR band at 407 nm (Supplementary Figure S3).

An increased SPR band intensity was observed after 5 and 8 h, along with a peak redshift (411 nm), indicating the progress of the reaction and the growth of the formed nuclei (Supplementary Figure S3). After 5 h, the particle size measured for AgHEC-T5 evidenced a slight increase, reaching 15 ± 5 nm; this change resulted in a denser population and a size distribution that shifted toward larger dimensions (Supplementary Figure S2C). For sample AgHEC-T12, sampled at 12 h, we observed a further increase in the density of population and a mean diameter of 17 ± 6 nm (Supplementary Figure S2). The white zones in the image correspond to the cellulosic agglomerates spread all around the sample. After 24 h, the SPR signal was noticeably increased in intensity, showing the maximum peak at a wavelength of 415 nm (Supplementary Figure S3). At this time, the reaction appeared almost complete, with a mean particle diameter of 19 ± 6 nm as measured by the FE-SEM image (Supplementary Figure S2E, F).

**FIGURE 2:**
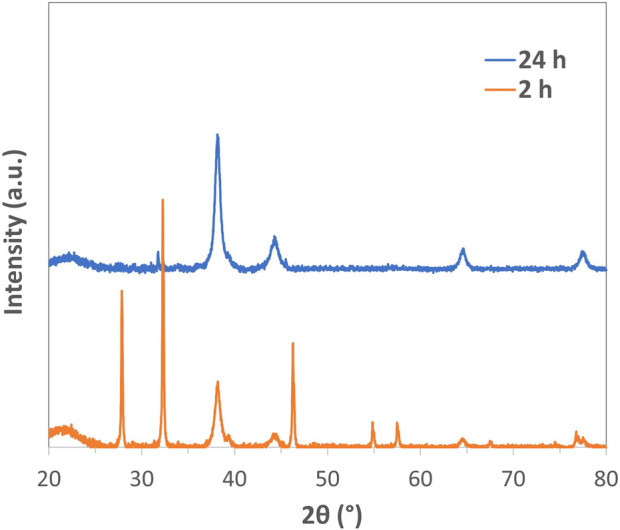
XRD spectra collected on sample AgHEC-01 after 2 and 24 h of reaction.

The progress of the reduction process promoting the formation of AgNPs was further followed through XRD measurements at 2 and 24 h of the reaction (Figure 2). After 2 h of the reaction, the data show well-recognizable peaks, confirming the formation of silver chloride (AgCl cubic phase, JCPDS No. 31-1238) as an intermediate product obtained after the addition of AgNO_3_ to the HEC solution, containing chloride counterions. After 24 h, the progress of the reduction reaction allowed us to obtain the metal phase, as pointed out by the peaks imputable to Ag (red curve, JCPDS No. 04-0783). The broad signals, typical of the Ag nanophase, revealed a mean crystal dimension of 15 nm, calculated by the Debye–Scherrer equation, and consistent with the mean particle size observed by TEM images.

### 3.2 Investigation of the design space synthesis options for multi-optimization

Different synthesis options depending on a selected set of independent synthesis variables offer design alternatives to maximize the functionality of the AgNPs. Indeed, the design space exploration has multiple objectives: (a) to investigate the implication of the synthesis parameters on the resulting AgHEC physicochemical features, (b) to assess the performance of AgHEC products associated with different synthesis options, and (c) to assess the toxicology and environmental impacts associated with the synthesis options, aiming for a safer and more sustainable synthesis process, while maximizing the antibacterial functional performance of the nano-silver product.

For this purpose, as the first step of the multi-optimization problem, we defined the synthesis design space by selecting two synthesis variables considered the main relevant factors in driving the reduction, nucleation, and growth mechanisms of nanoparticles. The selected variables were the molar ratios between the key reagents, HEC/Ag and NaOH/Ag, which were also believed to have major effects on the functional performance of the obtained nano-silver products.

For the second step of the multi-optimization process, we defined the range of variability for each molar ratio. This included variations in the HEC/Ag ratio between 1.4 and 6.4 and NaOH/Ag ratio between 1.4 and 3.7. We expanded the design space by exploring the conditions near AgHEC-01 corresponding to the standard procedure (HEC/Ag = 5.5; NaOH/Ag = 2.8). Within the two-dimensional decision space, a specific domain to be explored has been defined in order to control the nanoparticles’ size and identify a stability zone of the prepared colloidal nanodispersion.

Instead of full factorial DoE, a fractional factorial DoE model was devised to comply with the requirement for a minimum yet sufficient number of samples needed to quantify the addressed KPIs related to the functional, safety, and sustainability dimensions. The choice of the partial DoE, which implied generating n.6 different samples instead of n.9 samples, complies with the limitations imposed on the experimental burden, which required a proportional number of experimental assessments associated with the samples for all the addressed KPIs. In the third step, a set of synthesis variants was prepared to explore the design alternatives within the design space. This was achieved by independently changing the molar ratios between the key reagents, HEC/Ag and NaOH/Ag, within the defined variability range, and a design of experiment (DoE) matrix was generated.

The fourth step implied in the multi-optimization process is the generation of experimental and modeling data.

**FIGURE 3:**
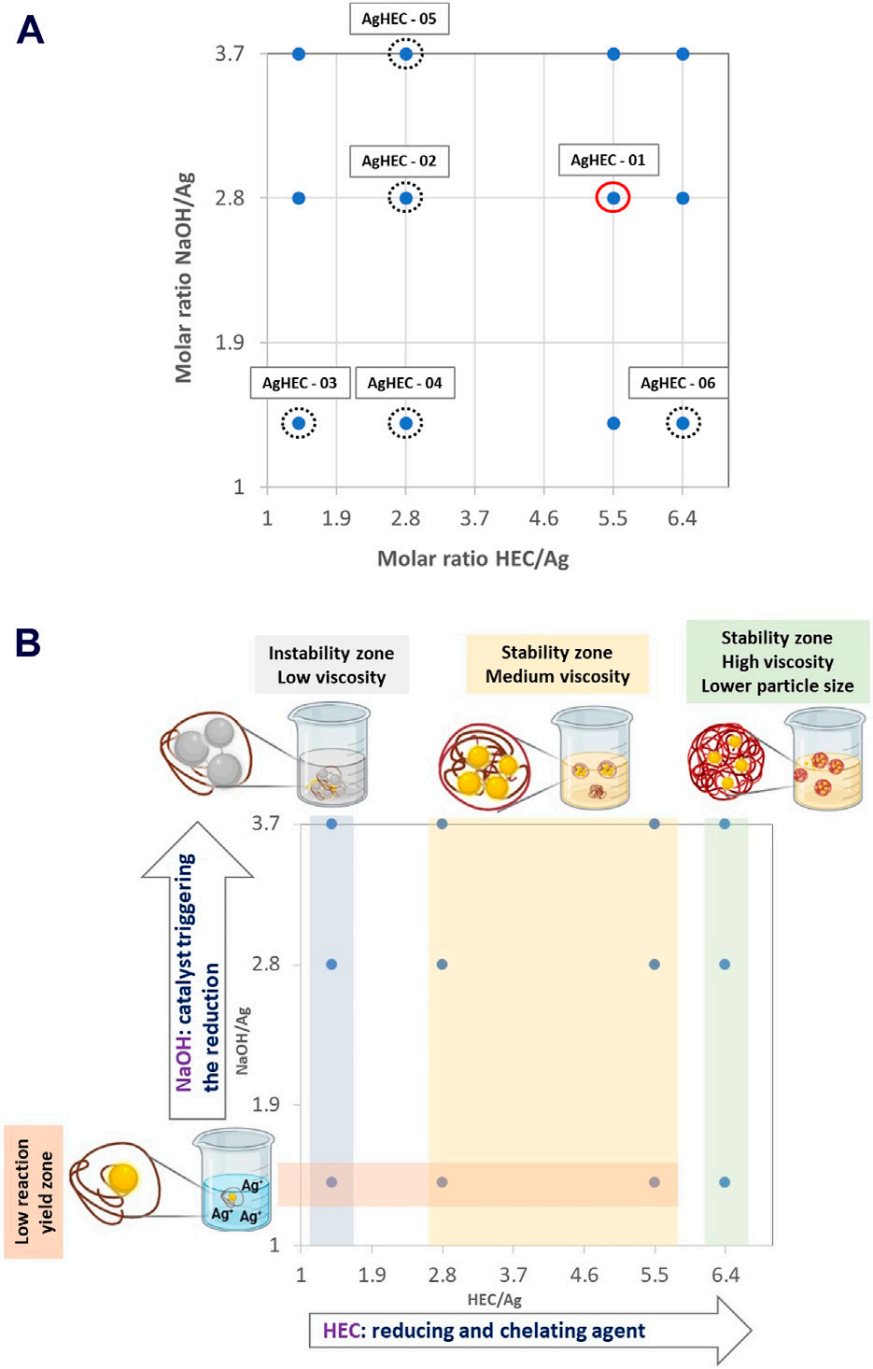
Design space defined for AgHEC combining two independent synthesis parameters (HEC/Ag and NaOH/Ag molar ratios): **(A)** DoE matrix and experimental points (red circles correspond to the standard procedure, and black-dotted circles correspond to the explored design alternatives); **(B)** identified design space zones sharing similar behavior.

Figure 3A highlights the points identified in the design space that correspond to the prepared samples (design alternatives). The resulting samples were overall characterized to evaluate how the synthesis parameters influence the physicochemical features (Table 1), and four zones of the design space sharing similar behavior in terms of colloidal stability, viscosity (discussed in Section 3.3), reaction yield, and particle size distribution have been identified (Figure 3B).

**FIGURE 4:**
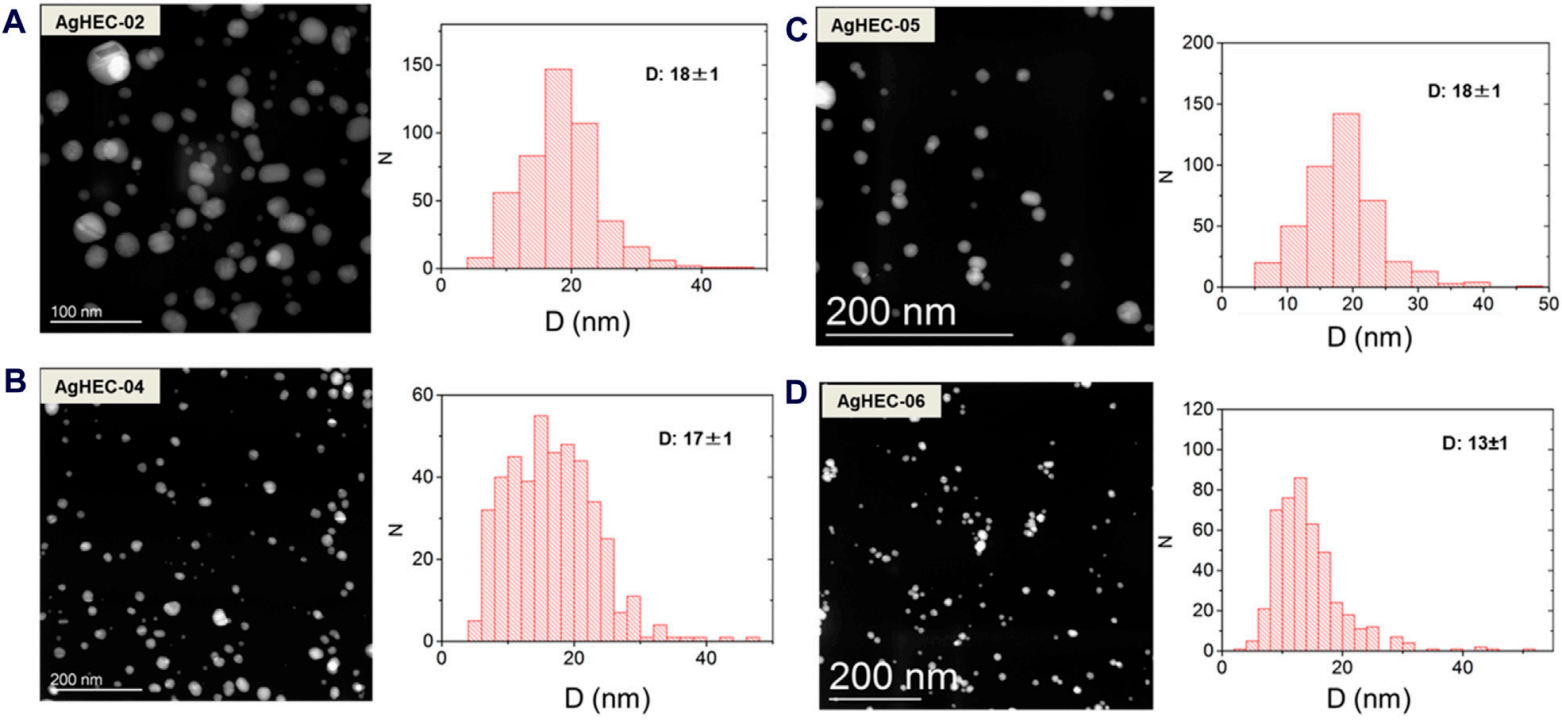
TEM images and corresponding particle size distribution measured on more than 400 NPs for the prepared AgHEC alternatives: **(A)** AgHEC-02; **(B)** AgHEC-04; **(C)** AgHEC-05; and **(D)** AgHEC-06.

Sample AgHEC-02, prepared by halving the stabilizing polymer content of AgHEC-01, showed a decreased hydrodynamic diameter (174 vs. 286 nm) due to a lower cellulose amount associated with a reduced steric hindrance of the polymer. For this sample, Z-potential pointed out a less positive value, consistent with a lowering of the positive surface charge provided by the HEC matrix. On the other hand, the reduced amount of HEC, acting as a chelating agent and able to control the growth step, produced particles slightly larger than AgHEC-01, as detailed by TEM images and size distributions (Figure 4A).

By further decreasing the cellulose loading for sample AgHEC_03, we observed an abrupt decrease in the colloidal stability, probably because at such HEC concentrations, the reducing/stabilizing action was insufficient to control the growth of the nanoparticles. For AgHEC-03, we assessed a hydrodynamic diameter of an order of magnitude higher than AgHEC-01 (3,280 nm), a Z potential value close to 0 (3.9 mV), a very high PDI (0.8), and a redshifted absorption peak (424 nm) confirmed by the UV–VIS spectrum (Supplementary Figure S4). Such features are consistent with the precipitation of large particles, observed by the naked eye as dark gray powder. Such a low HEC/Ag ratio involves a weak reducing power, driving the formation of a limited number of nuclei and promoting the growth stage over nucleation. As a result, we observed a poor overall reaction yield, which is aligned with the limited reducing power.

The formation of Ag nanoparticles is catalyzed in alkaline conditions. AgHEC-04 contains a halved amount of both HEC and NaOH, and the characterization results showed values of particle size (Figure 4B), hydrodynamic diameter, and Z-potential close to the data collected for the same HEC/Ag ratio (2.8), confirming that HEC affects both particle size and surface charge. However, AgHEC-04 evidenced a non-complete reaction yield (82%) consistent with the lower intensity of the SPR assessed by UV–VIS (Supplementary Figure S4). Such data emphasized the role of NaOH acting as a catalyst and being able to trigger the reduction reaction. Without adding NaOH, the reaction kinetic slows down significantly with a scarce conversion of ions to NPs (Chou et al., 2005; Watanabe et al., 2005; Blosi et al., 2014). The presence of NaOH also confers an alkaline pH to all the Ag-based suspensions (Table 1).

In sample AgHEC-05, prepared by maintaining the ratio HEC/Ag at 2.8, we increased the amount of the catalyst until an NaOH/Ag ratio of 3.7 was reached. At this condition, we reached a complete reaction yield. As expected, real particle size (Figure 4C), hydrodynamic diameter, and Z-potential are the physicochemical features mainly affected by the HEC component, showing values close to the samples prepared at the same HEC/Ag ratio (AgHEC-02 and AgHEC-04).

Increasing the polymer component until an HEC/Ag ratio of 6.4 is reached and keeping at 1.4 the NaOH/Ag ratio (AgHEC-06), we obtained nanoparticles with larger hydrodynamic diameter, due to the increased steric hindrance, and a more positive Z-potential. In this case, the higher amount of HEC, also acting as a reducing agent, balances the low NaOH content and enables it to reach a total reaction yield. The particle size measured by TEM revealed a mean diameter of 13 ± 1 nm, lower than that of the baseline sample (AgHEC-01). This value is consistent with a higher number of smaller nanoparticles with dimensions below 10 nm (Figure 4D), and it is probably driven by the chelating agent added in large excess and enabling an improved control of the growth step.

### 3.3 Viscosity study at different synthesis parameters

**FIGURE 5:**
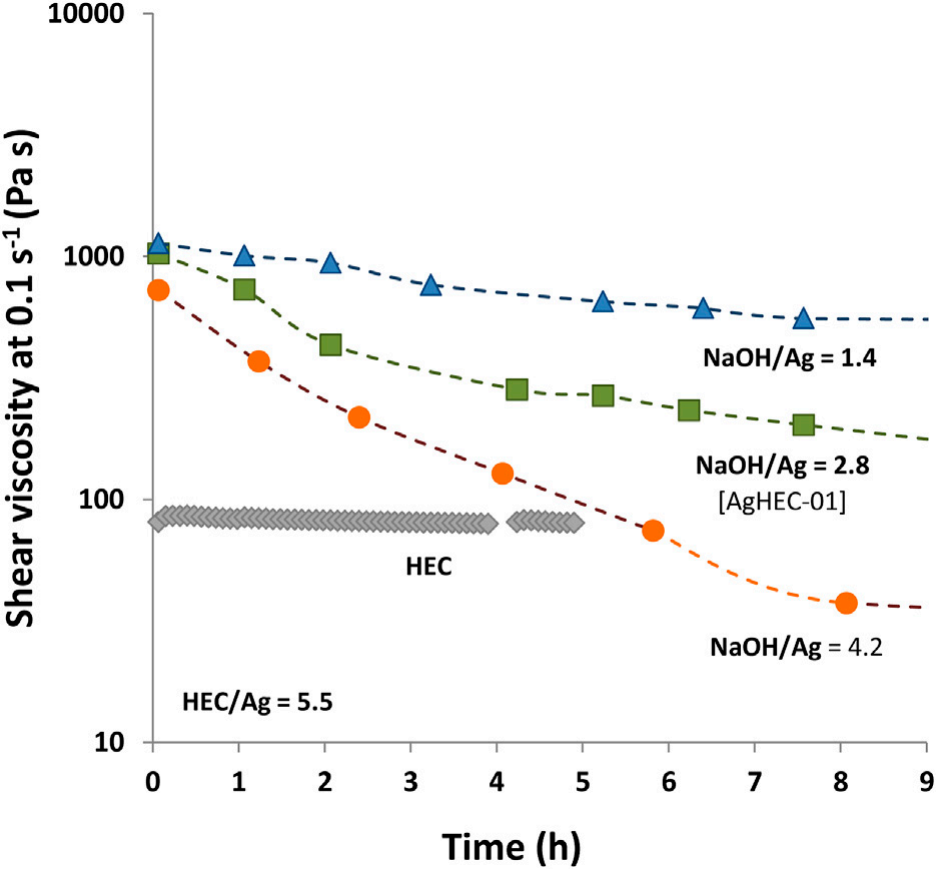
Viscosity measurements of AgHEC with different NaOH/Ag ratios (HEC/Ag molar ratio = 5.5 and silver concentration = 0.5 % wt). Viscosity of HEC without adding AgNO_3_ (gray curve).

HEC treated in an alkaline environment abruptly increases the viscosity of the nanosol until reaching the hydrogel form, which progressively turns into a less viscous suspension. Monitoring the viscosity over time allowed us to gather information on the reaction mechanism. Through tailored viscosity measurements, we evaluated the hydrogel consistency evolution, also visible to the naked eye, and related to the different arrangements of the HEC macromolecules in response to the evolution of synthesis stages. After adding the alkaline solution, we observed that HEC reacts by forming a viscous hydrogel that reduces the ionic diffusion. Nevertheless, the addition of the alkaline solution triggers the oxidation of the aldehydic groups, acting as a reducing agent for the Ag^+^ ions and promoting the formation of AgNPs. This results in a progressive de-structuring of the HEC hydrogel, observed within the first 24 h, as demonstrated by experimental measurements. Upon the addition of the NaOH solution, a high value of viscosity was found (approximately 1,000 Pa s), due to the formation of a cellulosic network, and then a decrease of viscosity over time (8 h), up to 200 Pa s, was observed (red curve in Figure 5). It is reasonable to assume that such a viscosity decrease is due to the hydrolysis of HEC combined with its oxidation to nucleate AgNPs (Figure 5). The control sample, prepared under alkaline conditions without silver precursors, showed a constant viscosity as a function of time (gray curve in Figure 5), and only very slow polymer hydrolysis was detected (Wolfenden et al., 1998).

**FIGURE 6:**
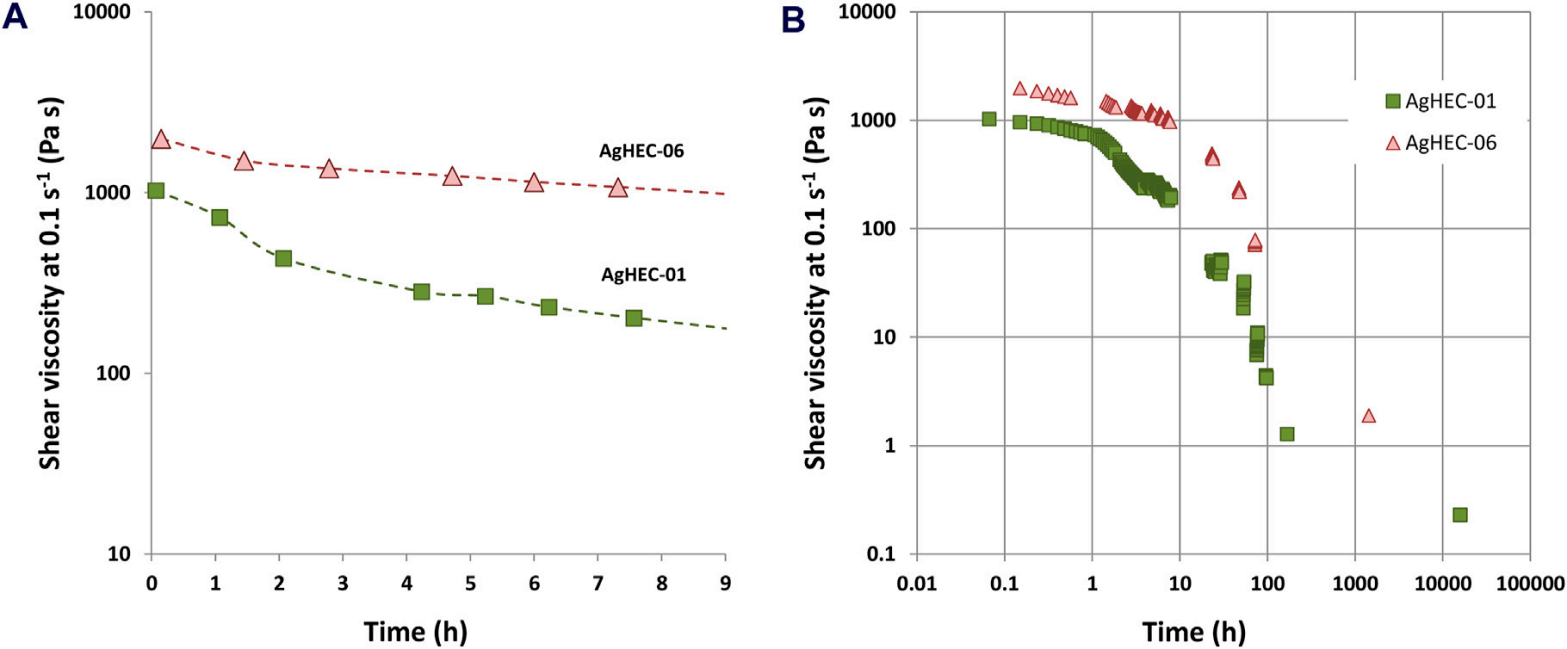
Viscosity measurements of AgHEC-01 and AgHEC-06 at **(A)** short (less than 8 h) and **(B)** long (till 22 months) times.

To further investigate the synthesis mechanism, a set of viscosity measurements was carried out on samples prepared by adding different amounts of NaOH. Particularly, the NaOH/Ag ratio was explored in the range between 1.4 and 4.2. Since NaOH acts as a catalyst for reduction, for an increased NaOH/Ag ratio, the reaction process takes place faster, also involving a faster viscosity decrease over time (Figure 5). Such behavior appears consistent with the pH-dependent hydrolysis of HEC that controls the kinetics of AgNP formation and the structure of the resulting hydrogel. The possibility of modulating the viscosity and, as a consequence, the kinetic of AgNP nucleation, based on the quantity of NaOH, paves the way for expanding the application field of AgHEC, allowing its long-term delivery, making this technology particularly appealing for applications such as antimicrobial additives or as coating of different supports (fabrics, ceramic tiles, steel, plastic materials, wound dressings, and biomedical devices). The possibility of releasing the antimicrobial agents in a sustained manner is indeed fundamental to treating infections effectively and preventing biofilm formation, with very interesting future perspectives (Ng et al., 2014). Furthermore, the amount of the HEC polymer contributes to modification of the viscosity of the hydrogel. As expected, AgHEC-06, prepared by increasing the HEC/Ag ratio to 6.4, has a higher viscosity value in comparison with AgHEC-01 (Figure 6A). The measurements repeated after long periods of time (till 2 months for AgHEC-06 and 22 months for AgHEC-01) show a strong reduction in viscosity for both the samples, well below the viscosity of HEC (approximately 80 Pa s), indicating a continuous degradation of the polymer interacting as a reducing and chelating agent with AgNPs (Figure 6B).

### 3.4 Antibacterial activity in suspension

We evaluated the antibacterial activity of the prepared AgHEC variants to identify how the different synthesis parameters affect the antimicrobial response of the prepared nanoparticles. Based on the results reported in Table 2, 10 ppm was identified as the minimum active concentration that can promote a bacterial reduction higher than 1 log and correspond to a percentage depletion higher than 90%.

**TABLE 2:** Antibacterial activity results collected on AgHEC-based samples. Each sample is reported together with the corresponding blank solution. Tested concentrations are referred to the Ag dose, and blank solutions are diluted accordingly.

Sample code	Log reduction* *E. coli*				Log reduction* *S. aureus*			
Concentration (mg/L)	50	10	1	0.5	50	10	1	0.5
AgHEC-01		1.19	<0.3	<0.30		0.62	<0.30	<0.30
HEC-01		1.43	0.90	<0.30		<0.30	<0.30	<0.30
AgHEC-02		1.82	0.47	<0.30		0.43	<0.30	<0.30
HEC-02		0.65	0.61	<0.30		<0.30	<0.30	<0.30
AgHEC- 04		1.15	<0.30	<0.30		1.65	<0.30	<0.30
HEC–04		1.39	<0.30	<0.30		<0.30	<0.30	<0.30
AgHEC-05		1.85	<0.30	<0.30		1.48	<0.30	<0.30
HEC-05		1.75	1.11	0.80		1.08	0.41	<0.30
AgHEC-06	>5.45	4.08	0.48	<0.30	4.64	3.53	0.56	<0.30
HEC-06		1.55	0.98	0.69		<0.30	<0.30	<0.30

No relevant differences were detected among the samples from AgHEC-01 to AgHEC-05, indicating that the different synthesis parameters poorly affect the final antibacterial activity. Furthermore, for these samples evaluated by using this testing method, the presence of AgNPs did not modify the activity with respect to the corresponding blank, resulting in the final performance very close to that of the HEC alone. As expected, blank solutions based on HEC only provided antimicrobial activity, with an efficacy aligned with that of AgHEC compounds if tested against *E. coli* and slightly reduced if tested against *S. aureus*. The positive charge associated with the polymer could be responsible for its improved activity against Gram-negative bacteria characterized by a higher negatively charged cell wall (Alfei and Schito, 2020).

It is worth highlighting the excellent activity measured for AgHEC-06, which pointed out a log reduction ranging from 3 to 4 (99.9%–99.99%) against both the tested bacteria at a concentration of 10 ppm and from 4 to 5.5 (99.99%–99.999%) when tested at 50 ppm (Table 2).

In this case, the blank activity showed values close to that of the other tested blanks and no effect against *S. aureus*, evidencing the key role of the inorganic phase and likely resulting in a synergistic effect promoted at this synthesis condition. We can hypothesize that the increased antimicrobial activity shown by AgHEC-06 could stem from the presence of a smaller particle fraction (Agnihotri et al., 2014; Dong et al., 2019; Korshed et al., 2019) in line with TEM images (Figure 4) that reveal an increased population in the range below 10 nm. Literature reports that the antibacterial efficacy of AgNPs is significantly correlated with the size, and generally the smaller the particle size, the higher the antibacterial activity. Several papers show that the antimicrobial activity of AgNPs gradually increases with the particle size decreasing from 100 to 5 nm and promoting an overall enhanced reactivity (Choi and Hu, 2008; Lu et al., 2013; Agnihotri et al., 2014). Despite the exact antibacterial mechanism of action not yet fully understood and still under debate, the presence of smaller nanoparticles implies an increased reactivity for all the varieties of hypotheses of action, such as the release of silver ions, the production of reactive oxygen species (ROS), and the mechanical damage due to the nanosized particle accumulation into the bacteria cell wall (Hayashi et al., 2013; Wang and Vermerris, 2016; Perinelli et al., 2018; Kukushkina et al., 2021).

### 3.5 Safety and sustainability profile of the synthesis process

The LCA and LCC analyses were carried out for the reference cases of the DoE matrix, allowing us to explore the design space and assess environmental sustainability and cost performance for the synthesis variants. The impact selected CML2001 impact method in LCA analysis provided a full environmental profile of all reference design cases addressed in the DoE matrix for all addressed LCA impact categories. Among the impact categories, two have been selected: climate change (GWP100) and the HTTP. These indicators are midpoint impact indicators: the first accounts for the environmental burden associated with the greenhouse effect and the second for the potential toxicity effects on human health. The metrics are expressed in kg-equivalents of carbon dioxide for the first impact category and in kg-equivalents of dichlorobenzene for the second one. Table 3 reports the computed values for the six AgHEC synthesis methods referred to in the DoE matrix, where sample AgHEC-03 (associated with the values HEC/Ag = 1.4 and NaOH/Ag = 2.8), has been replaced with sample AgHEC-03B (associated with the values HEC/Ag = 6.4 and NaOH/Ag = 3.7). The results from the costing analysis are also reported. The impact category selection allowed dealing with one KPI for environmental sustainability (GWP), one KPI for human safety (HTTP), and one economic KPI for cost-effectiveness.

**TABLE 3:** Harmonized dataset obtained from LCA and LCC reporting: environmental impact on climate change, impacts on human toxicity, and costs of each synthesis process related to the reference design cases of the DoE matrix.

Sample	HEC/Ag	NaOH/Ag	Climate change GWP100	Human toxicity HTTP	Cost/FU
	*(wt/wt)*	*(wt/wt)*	*(kg CO*_*2*_ *eq)*	*(kg DB eq)*	(€/FU)
AgHEC-01	5.5	2.8	0.0922	0.7400	0.30
AgHEC-02	2.8	2.8	0.0894	0.7383	0.25
AgHEC-03B	6.4	3.7	0.0932	0.7406	0.32
AgHEC-04	2.8	1.4	0.0893	0.7382	0.31
AgHEC-05	2.8	3.7	0.0895	0.7384	0.25
AgHEC-06	6.4	1.4	0.0930	0.7405	0.32

By combining the results of the antibacterial functionality tests, process yields, and results from the costing assessment, it was possible to create a compound key performance indicator intrinsically accounting for the expressed antibacterial functionality against both *E. coli* and *S. aureus* bacteria strains, referred to as the cost per functional unit produced in the synthesis process. This was done through the definition of a compound antibacterial activity (cAA) performance indicator by averaging the AA level for the two bacteria strains obtained for 10 mg/L concentrations of Ag-based suspensions. The resulting performance indicator values for the sample reference cases are reported in Table 4.

**TABLE 4:** Functionality compound performance indicators obtained by averaging the antibacterial level of each reference sample associated with the DoE matrix against *E.coli* and *S. aureus* bacterial strains. The compound antibacterial functionality is obtained by averaging the AA levels obtained at 10 mg/L silver dose concentrations. The compound functional quality factor is obtained by combining the compound antibacterial functionality indicator with the functional unit cost.

Sample	HEC/Ag	NaOH/Ag	Compound antibacterial functionality	Compound functional quality factor	Process yield
	*(wt/wt)*	*(wt/wt)*	*(cAA)*	*(cAA/€)*	*(%)*
AgHEC-01	5.5	2.8	0.91	3.00	99.99
AgHEC-02	2.8	2.8	1.13	4.41	100.00
AgHEC-03B	6.4	3.7	0.98	3.09	99.97
AgHEC-04	2.8	1.4	1.40	4.50	82.00
AgHEC-05	2.8	3.7	1.67	6.53	99.97
AgHEC-06	6.4	1.4	3.81	11.98	100.00

### 3.6 Multi-optimization for the safe and sustainable by the design synthesis process

Each point represented in the diagrams of Figure 8 belongs to the set of multi-optimal AgHEC synthesis design cases that simultaneously minimize specific environmental impacts and specific toxicity effects on human health, while providing the highest possible functional–economic performance by maximizing compound antibacterial activity against the two targeted bacterial strains while minimizing the production costs. Each point of the performance space is associated with one point in the design space specifying the synthesis variables and the physicochemical attributes of the nano-silver products. Based on the results provided by the MultiOptimal360™ platform, the safest and most sustainable by design synthesis process complying with addressed product functionality is the one corresponding to the sample AgHEC-06 and consistent with the circled point in the design space (HEC/Ag = 6.4 and NaOH/Ag = 1.4).

The choice of these synthesis factor expression of the sample AgHEC-06 provides a HTTP/FU = 0.084 kgDBeq/FU corresponding to 71% between the maximum and minimum value attainable for human toxicity, GWP = 0.093 kgCO_2_ eq/FU corresponding to 31% level between the maximum and minimum value attainable for climate change impacts, and cAA/cost = 11.98. cAA/€ corresponding to the maximum attainable compound functionality/cost level and the highest AA level attainable for *E. coli* (AA = 4.08) and *S. aureus* (AA = 3.53), respectively. The values of the synthesis factors are associated with the lowest value of TEM diameter (*ϕ* = 13 nm), the highest Z-potential (Z-pot = 20.1 mV), and a value of pH = 9.58, corresponding to a 29% level between the minimum and maximum values attainable.

**FIGURE 7:**
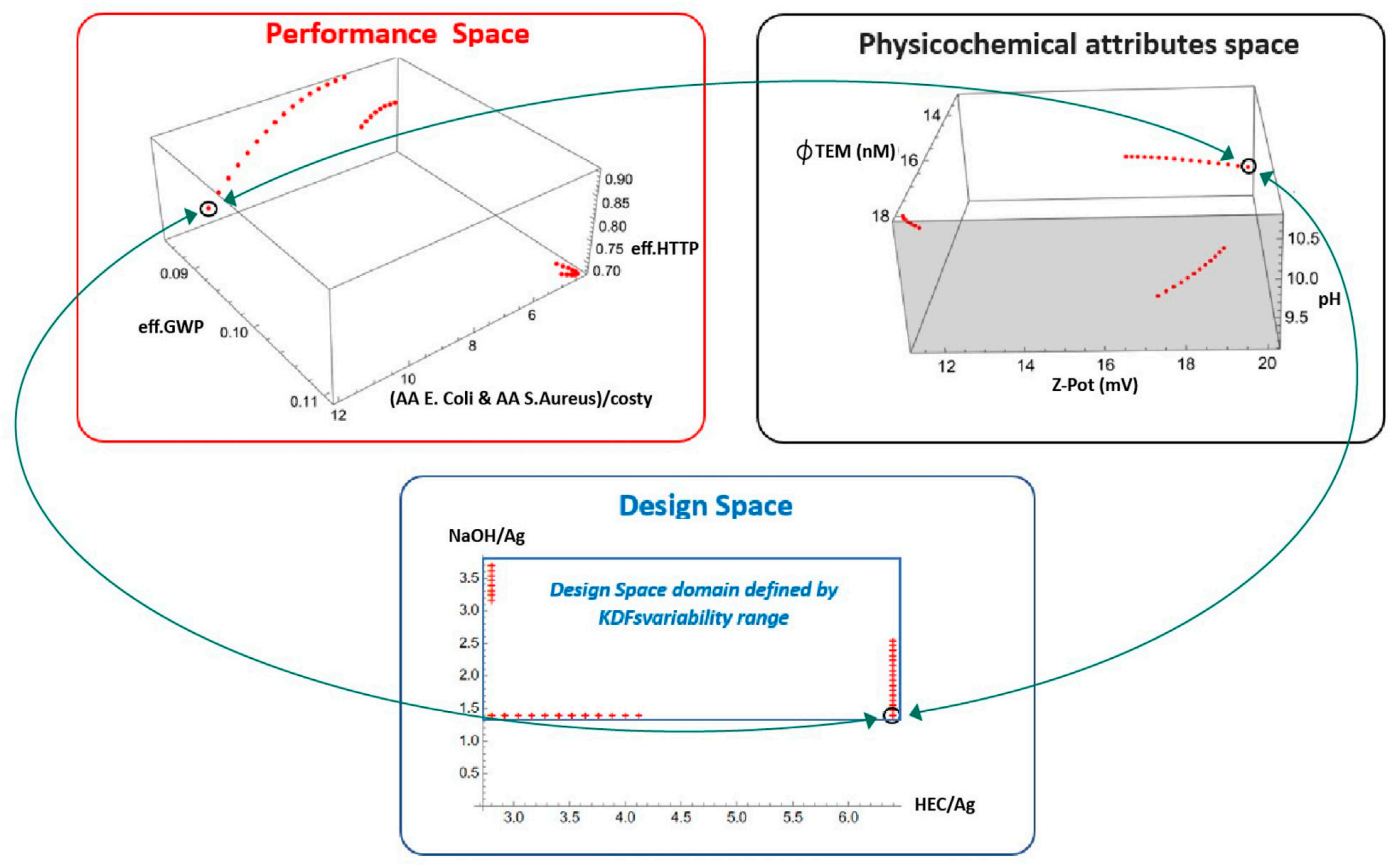
Multioptimal design cases represented in the design space, performance space, and physicochemical attribute space. Each point in the diagrams belongs to the set of multioptimal AgHEC synthesis design cases that simultaneously minimize environmental impacts, toxicity effects, and costs while maximizing functional–economic performance.

The values for the chosen synthesis factors (circled points of Figure 7) are the best ones for the associated functionality level and still comply with low toxicity and environmental impacts. The selection of different synthesis alternatives within the multi-optimal design cases would have unbalanced the performance of at least one of the addressed KPIs. Indeed, for instance, requiring the absolute lowest toxicity level would turn into an inconvenient choice as this is associated with a maximum environmental impact on climate change and the lowest functionality level (that would not comply with the minimum acceptable antibacterial activity level for both bacteria strains). Conversely, requiring the lowest environmental impact implies accepting the highest toxicity level and exceedingly low antibacterial functionality level.

### 3.7 Antibacterial activity of coated fabrics

Protection and preservation of fabrics from microorganisms have long played a crucial role and remain fundamental in many textile applications today, particularly in the hospital or medical environments suffering from the high concentration of pathogenic microorganisms strictly correlated with the incidence of nosocomial infections (Hiwar et al., 2021). Cellulose-based matrices, like HEC macromolecules, represent a suitable coating for natural and synthetic fibers (Kale et al., 2016a; Souza et al., 2022), favoring the grafting of nanoparticles on several types of substrates (Kale et al., 2016b; Fernandes et al., 2022). To verify whether AgHEC can vehiculate the antibacterial functionality to the nano-enabled products, we evaluated the antibacterial activity of nonwoven cellulose substrates coated by AgHEC-01 (C-01) and AgHEC-06 (C-06). The antibacterial activity of prepared samples pointed out an improved performance for C-06, which ensured a complete bacterial depletion for both tested bacteria, *E. coli* and *S. aureus*. Despite nanosuspensions being applied with the same metal content (0.01% wt), ICP-OES measurements showed an increased Ag loading for sample C-06, probably due to the higher amount of hydroxyethyl cellulose matrix included in AgHEC-06, which could trigger an improved transferring of NPs onto the fabric surface. Such improved metal transferring (11.0 μg/g for C-01 versus 14.6 μg/g for C-06) could be responsible for the higher antimicrobial performance detected for sample C-06 (Table 5). However, the marked difference in activity against *S. aureus* between the samples, 0% for C-01 and 100% for C-06, appeared consistent with the highest antibacterial response measured in suspension for AgHEC-06 and likely linked to its lower particle dimensions, as assessed by TEM images.

**TABLE 5:** Results of the antibacterial activity measured on nonwoven cellulose fabrics coated by AgHEC-01 and AgHEC-06.

Fabric sample	Applied sol *(0.01% wt)*	Bacterial depletion %		Ag loading (μg/g)
		*E. coli*	*S. aureus*	
Uncoated	—	0	0	**—**
C-01	AgHEC-01	47	0	11.0 ± 1.0
CBlank-01	HEC-01	32	0	**—**
C-06	AgHEC-06	100	100	14.6 ± 0.6
CBlank-06	HEC-06	9	19	**—**

**FIGURE 8:**
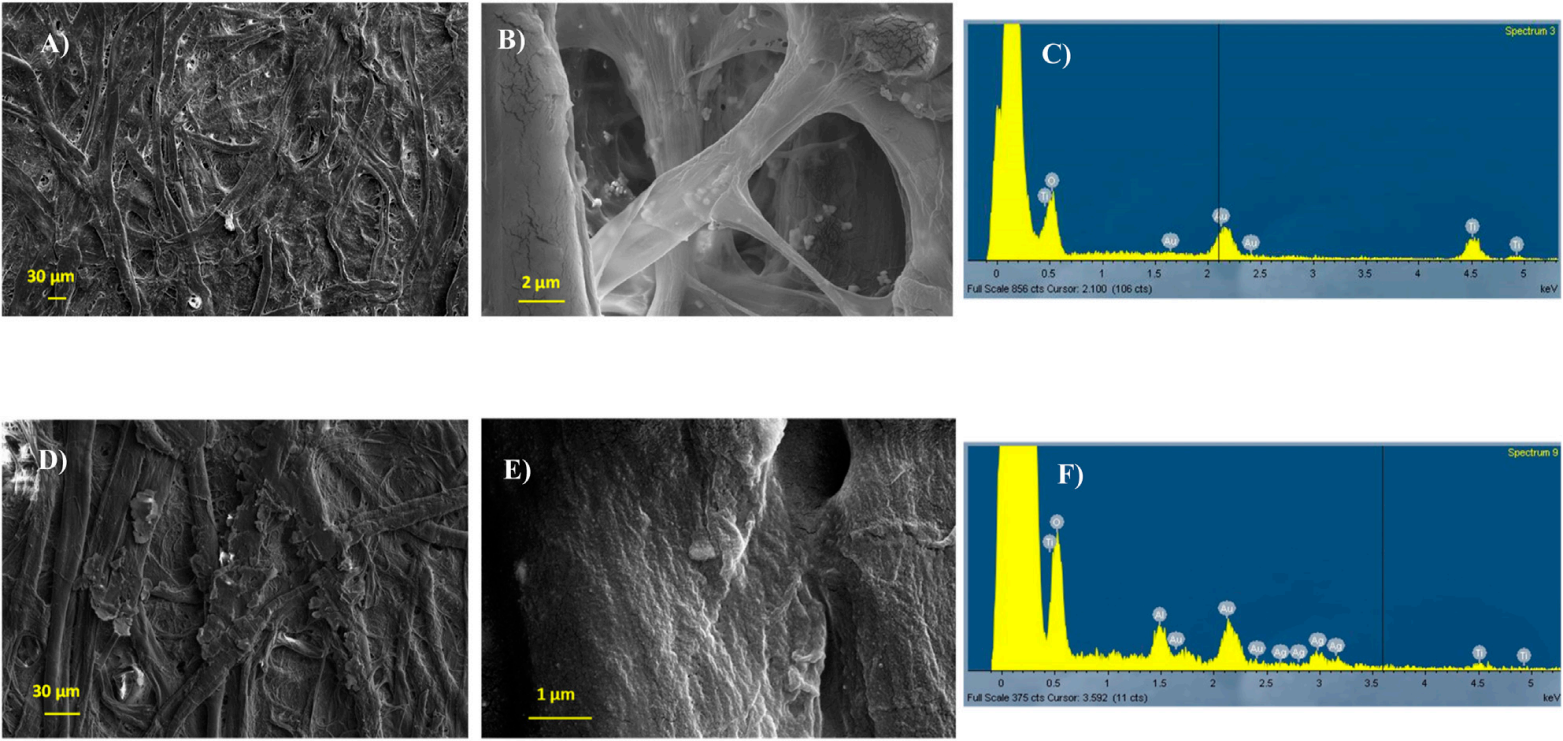
FE-SEM-EDX analysis of the fabrics: **(A)** uncoated sample at a low magnification; **(B)** uncoated sample at a high magnification; **(C)** EDX spectra collected on the uncoated sample; **(D)** AgHEC-01-coated sample at a low magnification; **(E)** AgHEC-01-coated sample at a high magnification; **(F)** EDX spectra collected on the AgHEC-01-coated sample.

FE-SEM images of the uncoated substrate highlighted a fibrous microstructure typical of nonwoven fabrics (Figures 8A, B). EDX signals collected on the uncoated surface revealed the presence of gold, added to ensure the sample conductivity, and titanium, consistent with the TiO_2_ rutile white pigment embedded into the fibers (Figure 8C). The sample coated by AgHEC-01 at a batch concentration of 0.01% wt pointed out the presence of plaques on the fibers (Figure 8D), probably produced by the HEC agglomeration on some surface points and due to the rapid water evaporation that occurred during the heating treatment. The images collected at higher magnifications on the fibers showed the presence of a nanostructure, consistent in size with AgNPs (Figure 8E). EDX spectra collected on different areas confirmed the presence of Ag as an element homogenously distributed on the fibers (Figure 8F).

## 4 Conclusion

We successfully developed a novel eco-friendly one-pot synthesis that allowed us to obtain Ag-based hydrogels at room temperature by a quick and easily scalable method. The process offers the chance to modulate the viscosity of the final AgHEC suspension and the kinetics of AgNP nucleation, paving the way for different engineering strategies for antimicrobial applications (additive, coating, and scaffolds). We exploited as a key benign reagent a quaternary ammonium salt of HEC with intrinsic antimicrobial activity acting as a chelating and reducing agent and emphasizing the antimicrobial effect in synergy with the inorganic counterpart (AgNPs).

The formation of AgNPs has been step-by-step monitored by an extensive physicochemical characterization, pointing out the formation of AgNPs reaching the completion after 24 h in the form of a stable and highly concentrated hydrogel with a potential tunable viscosity. A hypothesis of reaction has been advanced, and we explored a wider design space by changing two main independent variables (HEC/Ag and NaOH/Ag molar ratios) driving nucleation and growth steps.

The results confirmed that HEC mainly affects particle size, hydrodynamic diameter, and Z-potential, showing similar values for all the samples prepared at the same HEC/Ag ratio. A minimum amount of HEC is also fundamental to ensure an optimal reduction potential and achieve a total reaction yield. NaOH acts as a catalyst for reduction, triggering the reaction kinetics and involving a faster viscosity decrease promoted by contemporary HEC hydrolysis and oxidation.

The antibacterial activity evaluated in suspension highlighted an outstanding performance for AgHEC-06, the sample prepared with an excess of HEC and associated with an increased bacterial depletion compared to the blank and the other design alternatives. We hypothesized that the activity detected for AgHEC-06 stems from the presence of smaller particles, with a more populated fraction in the range below 10 nm and generated by the synthesis procedure with an excess of HEC. We also verified that AgHEC-06 ensured a total bacterial depletion even once immobilized on the substrate, confirming improved activity, if compared to the AgHEC standard formulation and increasing the Ag deposition on the fibers, probably favored by the high HEC amount.

The application of the multi-optimization process employing MultiOptimal360™ allowed the identification of the safest and most sustainable alternative within the identified decision space and confirmed AgHEC-06 (corresponding to the molar ratios of HEC/Ag = 6.4 and NaOH/Ag = 1.4) as the best design alternative.

## Data Availability

The original contributions presented in the study are included in the article/Supplementary Material; further inquiries can be directed to the corresponding authors.
