# Supplementary material for: Re-designing nano-silver technology exploiting one-pot hydroxyethyl cellulose-driven green synthesis
Source: Front Chem. 2024 Aug 14;12:1432546. doi: 10.3389/fchem.2024.1432546 (PMC11349673; doi:10.3389/fchem.2024.1432546)
Supplement: Supplementary file 1 [file DataSheet1.PDF]

Supplementary Material

Re-designing nano-silver technology exploiting one-pot hydroxyethyl cellulose-driven green synthesis

Blosi M[1§]*, Brigliadori A[1§], Ortelli S[1], Zanoni I[1], Gardini D[1], Vineis C[2], Varesano A[2,] Ballarin B[3], Perucca M[4], Costa AL[1]*

[1]*National Research Council of Italy, Institute of Science, Technology and Sustainability for Ceramics, (CNR-ISSMC) Faenza (Ra), Italy*

[2]*National Research Council of Italy, Institute of Intelligent Industrial Technologies and Systems for AdvancedManufacturing (CNR-STIIMA), Biella, Italy*

[3] Department of Industrial Chemistry *"Toso Montanari" Via Gobetti 85, Bologna, Italy*

[4] *Project HUB-360, 10051 Avigliana, Italy*

[§]*: These authors share first authorship*

*** Correspondence:** magda.blosi@issmc.cnr.it; anna.costa@issmc.cnr.it

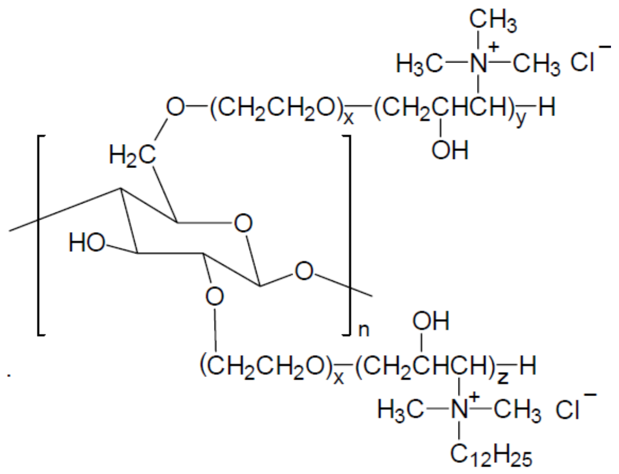

Figure S1 – Molecular structure of SoftCat SL30 (INCI Name: Polyquaternium-67) the quaternary ammonium salt of hydroxyethyl cellulose used as a chelating and reducing agent for the preparation of AgHEC.

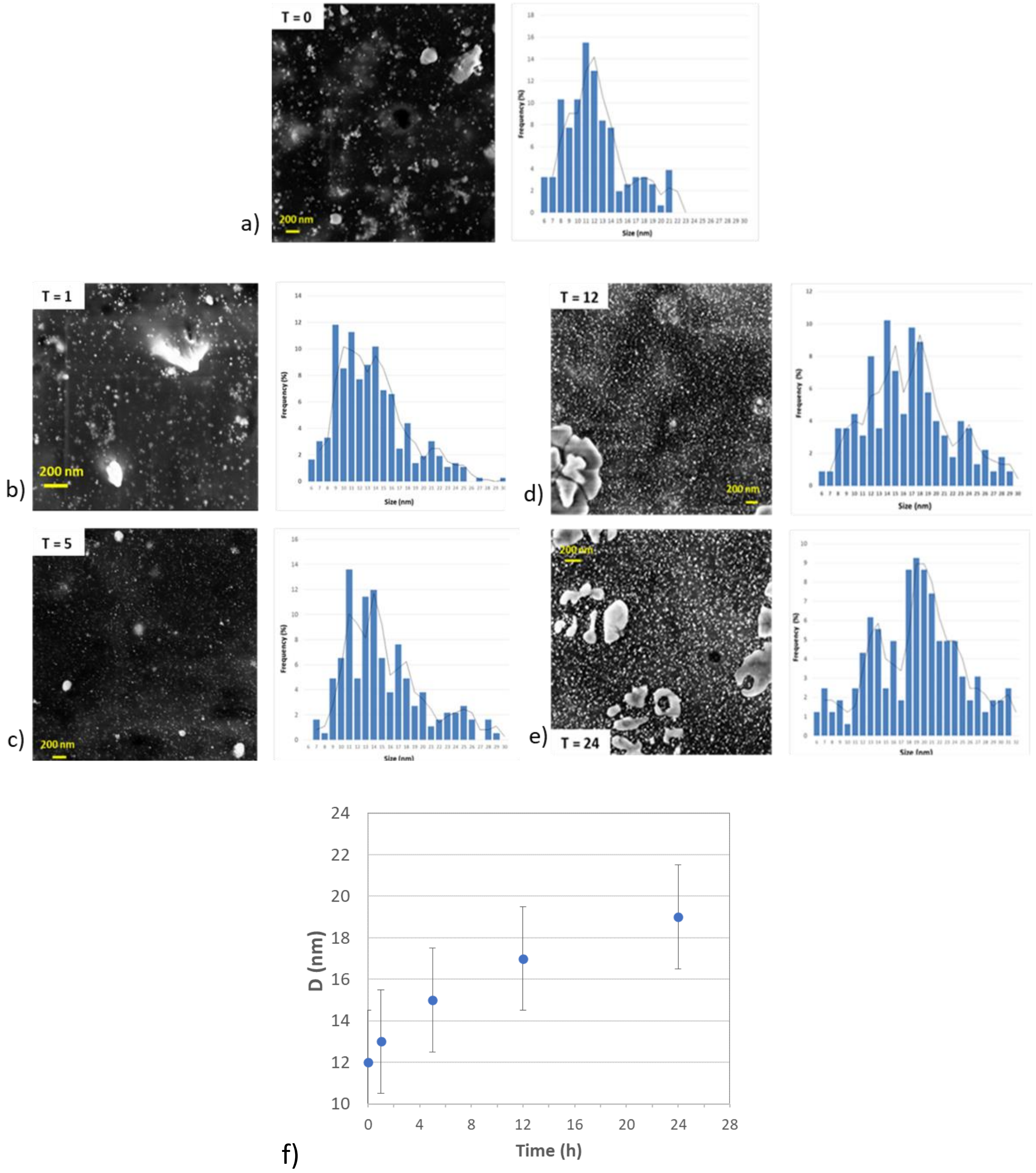

Figure S2 – FE-SEM images and corresponding particle size distribution measured on more than 150 NPs for AgHEC-01 at increasing reaction times: a) 0 h, b) 1 h, c) 5 h, d) 12 h, e) 24 h; f) graph representing the increase of the mean particle size derived by FE-SEM images.

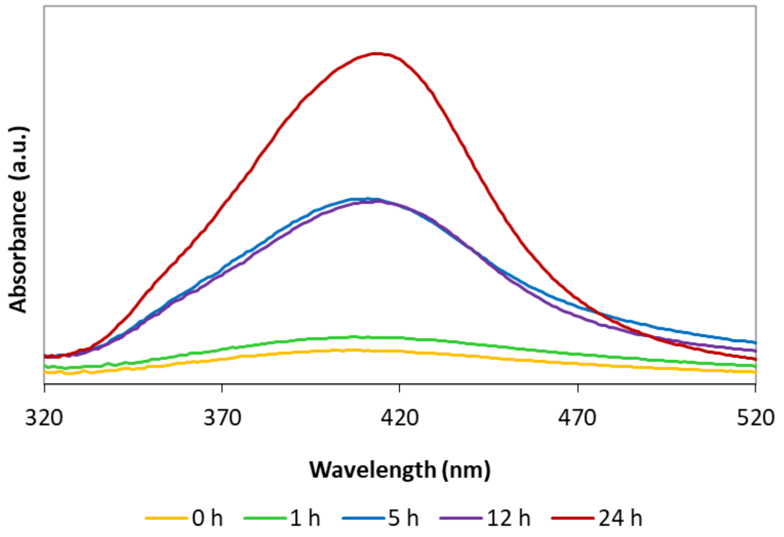

Figure S3 – UV-VIS spectra collected for sample AgHEC-01 at increasing reaction times: 0, 1, 4, 8 and 24 hours.

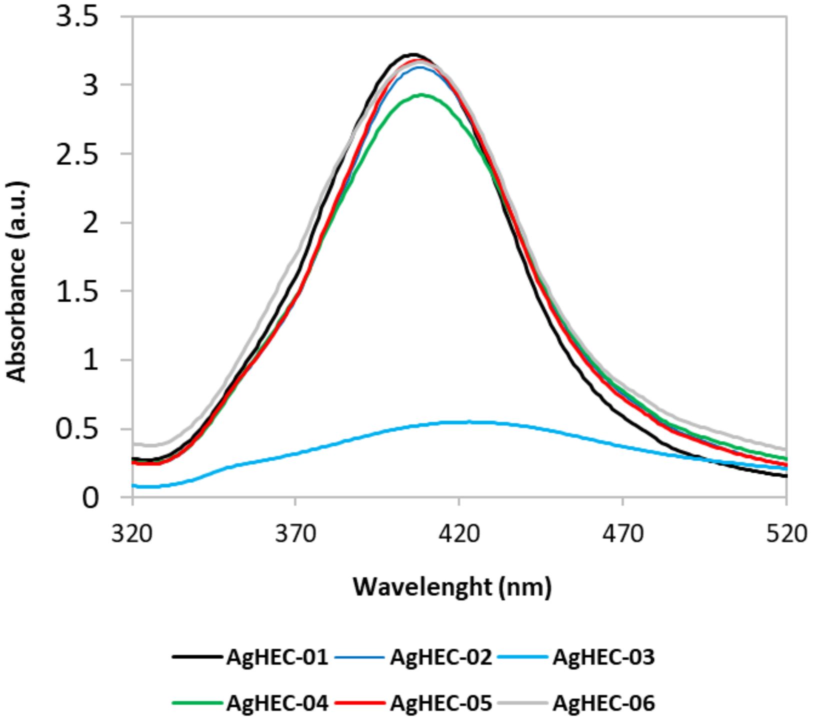

Figure S4 – UV-VIS spectra collected on AgHEC-based samples prepared at different molar ratios.
